# *Chlamydia trachomatis* Screening and Treatment in Pregnancy to Reduce Adverse Pregnancy and Neonatal Outcomes: A Review

**DOI:** 10.3389/fpubh.2021.531073

**Published:** 2021-06-10

**Authors:** Kristina N. Adachi, Karin Nielsen-Saines, Jeffrey D. Klausner

**Affiliations:** ^1^Division of Infectious Diseases, Department of Pediatrics, University of California, Los Angeles (UCLA) David Geffen School of Medicine, Los Angeles, CA, United States; ^2^Division of Disease Prevention, Policy and Global Health, Department of Preventive Medicine, University of Southern California Keck School of Medicine, Los Angeles, CA, United States

**Keywords:** *Chlamydia trachomatis*, sexually transmitted infections, pregnancy, infant outcomes, adverse pregnancy outcomes

## Abstract

*Chlamydial trachomatis* infection has been associated with adverse pregnancy and neonatal outcomes such as premature rupture of membranes, preterm birth, low birth weight, conjunctivitis, and pneumonia in infants. This review evaluates existing literature to determine potential benefits of antenatal screening and treatment of *C. trachomatis* in preventing adverse outcomes. A literature search revealed 1824 studies with 156 full-text articles reviewed. Fifteen studies were selected after fulfilling inclusion criteria. Eight studies focused on chlamydial screening and treatment to prevent adverse pregnancy outcomes such as premature rupture of membranes, preterm birth, low birth weight, growth restriction leading to small for gestational age infants, and neonatal death. Seven studies focused on the effects of chlamydial screening and treatment on adverse infant outcomes such as chlamydial infection including positive mucosal cultures, pneumonia, and conjunctivitis. Given the heterogeneity of those studies, this focused review was exclusively qualitative in nature. When viewed collectively, 13 of 15 studies provided some degree of support that antenatal chlamydial screening and treatment interventions may lead to decreased adverse pregnancy and infant outcomes. However, notable limitations of these individual studies also highlight the need for further, updated research in this area, particularly from low and middle-income settings.

## Introduction

Although *Chlamydia trachomatis* accounts for nearly 130 million new cases worldwide, it remains a perpetually overlooked global health issue, particularly in low and middle-income countries with limited resources (1–5). The consequences are exacerbated for pregnant women, where infection may be detrimental to the health of both mothers and their infants (3, 6).

Chlamydial infection in pregnancy has been associated with complications including fetal loss, premature rupture of membranes, preterm labor and delivery, and low birth weight among others (1, 3, 7–18). In particular, many studies, including a 12-study meta-analysis by Silva et al. (19), have found an association between chlamydial infection in pregnancy and increased risk for preterm labor, low birth weight, and/or perinatal mortality (12, 13, 15, 20). Some have suggested that untreated chlamydial infection in pregnancy may lead to as much as a two- to four-fold increased risk for preterm labor and delivery (12, 15, 20). The increased risk for preterm delivery with *C. trachomatis* infection is of particular concern given the high neonatal morbidity and mortality associated with premature birth (21). In addition, maternal infection with *C. trachomatis* may lead to neonatal infection including conjunctivitis and pneumonia due to high rates of vertical transmission, which some have estimated rates as high as 50–70% without treatment (22–25). It has been estimated that 30–50% of infants whose mothers have active, untreated *C. trachomatis* infection will develop conjunctivitis, and 10–20% of infants will develop pneumonia (11–13, 22).

Since *Chlamydia trachomatis* is an easily curable infection, antenatal screening programs that identify and treat infected mothers could potentially prevent many of these pregnancy and neonatal complications. The current U.S. Centers for Disease Control and Prevention (CDC) guidelines recommend a single dose azithromycin as first line treatment for chlamydia in pregnancy, with a course of amoxicillin or erythromycin listed as an alternative options (26, 27). Some have suggested that macrolides, such as erythromycin and azithromycin, may also be beneficial in preventing adverse pregnancy outcomes by suppressing tumor necrosis factor (TNF)-alpha, which has been implicated as an accomplice in preterm labor induction (28). Antenatal chlamydial screening and treatment for pregnant women, especially those considered high risk who are <25 years of age or with other risk factors, has been implemented in some countries such as the U.S, where it has been credited by some as the only effective means of preventing neonatal chlamydial infections (22, 29). While chlamydial screening and treatment of pregnant women was initially recommended by the CDC in the early 1980s, widespread implementation did not occur until the following decade (22). Some recent published studies have suggested the benefit of such interventions in the US by comparing rates of CT (pediatric seroprevalence and CT neonatal conjunctivitis) before and after routine implementation of CT screening and treatment in pregnant women in the US in 1993 (30, 31). These considerations are also important because studies have highlighted that standard neonatal ocular prophylaxis measures do not effectively prevent neonatal chlamydial conjunctivitis (32, 33).

Nevertheless, these initiatives remain controversial in many other countries (29). Both a 2016 Cochrane and 2014 USPSTF (US Preventive Services Task Force) systematic review of chlamydia screening have highlighted the lack of research investigating the benefits of chlamydia screening and treatment in pregnancy (34, 35). However, recent published studies from the US, Australia, and Netherlands have demonstrated that such interventions can be cost-effective in preventing morbidity associated with chlamydial infections, particularly among younger pregnant women in regions where chlamydia prevalence is high (36–38).

To comprehensively understand the potential benefits of an antenatal *Chlamydia trachomatis* screening and treatment intervention in pregnancy, a focused review of literature was performed. The specific objective was to review literature regarding the efficacy of screening and treatment interventions for *Chlamydia trachomatis* in pregnancy in preventing adverse pregnancy and neonatal outcomes.

## Methods

### Search Strategy and Selection Criteria

Primary searches using PubMed were initially completed on October 20, 2014 and subsequently updated three times on July 27, 2015, January 28, 2016, and July 30, 2016. An additional search was repeated in January 2020 to determine if additional published studies warranted inclusion. Searches were restricted to articles pertaining to humans, and articles published after 1970, which coincided with the first studies supporting vertical transmission of *Chlamydia trachomatis* from mothers to infants. We used broad search terms and many publications were ultimately excluded after review. We also assessed the reference lists of included studies and review articles for other relevant studies that may have been overlooked based on search criteria implemented. This process led to a review of all proceedings from the *International Symposium on Human Chlamydial Infections* in 1982, 1986, 1990 not available to PubMed, resulting in the inclusion of one additional study (39). PubMed search terms included “chlamydia AND pregnancy AND treatment,” “chlamydia AND adverse outcomes,” “chlamydia AND prematurity,” and “chlamydia AND neonatal infection,” which yielded a total of 1,824 articles. After restricting the search to only human studies and those published after 1970, 1,574 studies remained.

Preliminary screening was done based on titles of articles to exclude 1,245 articles that did not pertain to this review. Examples included a lack of focus on *C. trachomatis* in pregnancy, emphasis on other STIs or *Chlamydia* groups, and concentration on ectopic pregnancy and infertility complications. Three hundred twenty-nine abstracts were selected to be reviewed by one reviewer. Of those abstracts, 24 were excluded as they were not published in English. There were only two articles within available abstracts in English that could have potential relevance [Nishimura 1990 (Japanese) and Ottesen 1996 (Danish)] (40, 41). Other manuscripts excluded from full-text review included those focused only on screening, those without any adverse neonatal or pregnancy adverse outcomes reported, and those with only drug adverse outcomes reported such as potential congenital malformations. Because this review was not focused on discussing ectopic pregnancy, infertility, and other maternal adverse pregnancy outcomes such as chorioamnionitis, or post-partum endometritis potentially associated with chlamydial infection, articles that focused on these adverse pregnancy outcomes were also excluded.

### Assessment and Data Extraction

**Figure 1:**
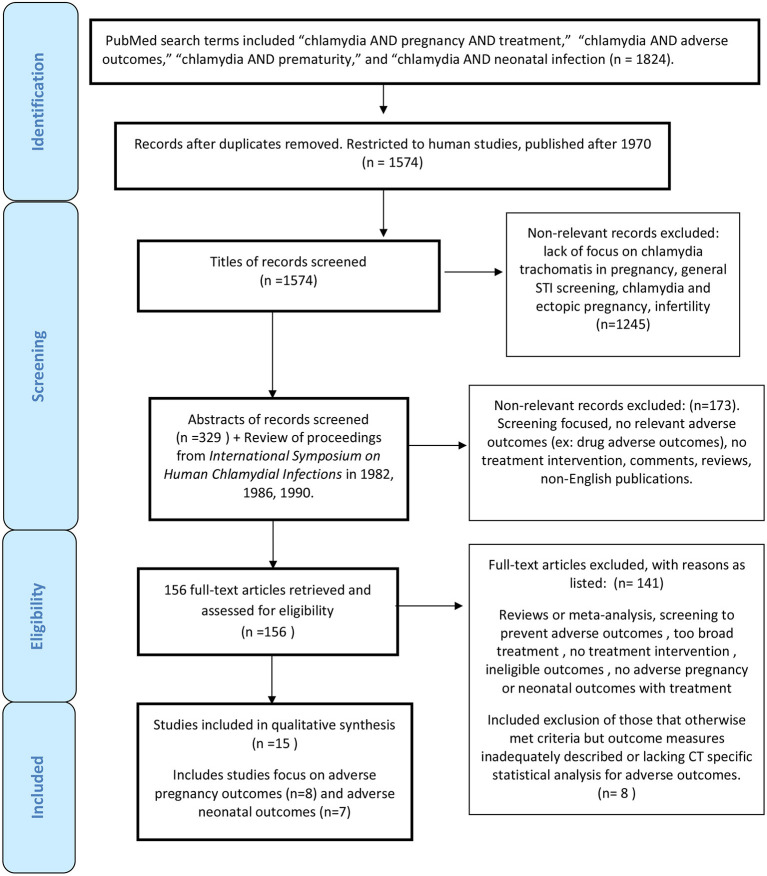
Flow diagram for chlamydial screening and treatment in pregnancy study inclusion.

One hundred and fifty-six documents were retrieved and evaluated in full-text review. Those that were considered for inclusion were classified as containing (1) treatment interventions in pregnancy with adverse pregnancy outcomes such as preterm birth, premature rupture of membranes, low birth weight, small for gestational age, or neonatal death; (2) treatment interventions in pregnancy with neonatal outcomes related to chlamydial infection such as positive chlamydial mucosal cultures, conjunctivitis or pneumonia. The 30 review articles were evaluated for other relevant articles that might have not been included in initial PubMed searches. Other articles did not meet study inclusion criteria due to lack of screening and treatment interventions or no neonatal or adverse pregnancy outcome measures with treatment intervention. Apart from the Cochrane Review (42), there were two articles (43, 44) that otherwise met inclusion criteria, but outcome measures were not adequately described. Another three articles lacked any CT specific analysis with respect to adverse outcomes (45–47). Two other recent studies evaluated rates of neonatal and pediatric CT pre- and post- routine implementation of CT screening and treatment in pregnancy in the US but were excluded given lack of information about specific maternal screening and treatment implemented in both and lack of CT specific neonatal outcomes in one, only positive CT serology in children under 10 years of age [(30, 31); Supplementary Table 1]. Our review was compliant with the PRISMA checklist for systematic reviews [(48); Figure 1].

## Results

### Antenatal Chlamydial Screening and Treatment to Prevent Adverse Pregnancy Outcomes and Neonatal Chlamydial Infection

While there is a large body of literature investigating the treatment of chlamydial infections in pregnancy, few of these studies provide meaningful data relevant to this review. The majority of those existing treatment studies focus on adverse treatment outcomes pertaining to drug safety and tolerability and test of cure (42).

Of the 15 studies that have been included in this review, eight of the studies provided at least some information on the chlamydial screening and treatment effect on adverse pregnancy outcomes such as low birth weight, preterm delivery, preterm labor, or premature rupture of membranes; the other seven studies provided at least some information on the chlamydial screening and treatment effect on neonatal chlamydial infections^1^. Of note, the Cochrane Review has been intentionally excluded from the 15 primary studies included in this review (42). While it was a meta-analysis of 11 randomized controlled trials for chlamydial treatment in pregnancy, few of the studies included evaluated adverse pregnancy or neonatal outcomes; it only featured 2 studies (Alary et al. and Bell et al.) discussed in our adverse neonatal outcomes section and 1 study (Martin et al.) discussed in the adverse pregnancy outcomes section [(39, 42, 49, 50); Refer to Supplementary Table 1 for further detail].

These two major outcome groups (pregnancy outcomes and neonatal outcomes) will be discussed separately in this review. Evaluation of the studies in both groups were not amenable to meta-analysis given the limited number of studies in each group as well as the different study designs, study objectives, variable study outcome measures employed. Thus, this paper exclusively focuses on a qualitative analysis. With regards to adverse pregnancy outcomes, focus is placed on those pertaining to infant health outcomes, specifically preterm delivery and other relevant measures including preterm premature rupture of membranes, premature rupture of membranes, preterm labor, and low birth weight.

### Studies Preventing Adverse Pregnancy Outcomes

#### Study Characteristics

We evaluated eight studies (5, 28, 50–55) that provided information regarding the effect of chlamydial screening and treatment in preventing adverse pregnancy outcomes. All of these studies were written in English; six of the studies were conducted in the U.S., and the others took place in Uganda and in India (28, 53). All of the studies in the U.S. focused primarily on non-white, young women (5, 50–52, 54, 55). The studies included primarily young, black women (5, 50–52, 55). Four studies mentioned that women were of lower socioeconomic status (5, 51, 52, 55), but only four commented on either alcohol (50, 54), smoking (5, 50, 52, 54), and/or illegal substance use (54). Only one study focused on HIV-infected pregnant women (28). Studies ranged in publication from 1990 to 2014. Initial cohort sizes ranged markedly based on study objective, enrollment ranged from 229 to 13,750 women [(52, 54); Table 1].

**Table 1:** Prevention of adverse pregnancy outcomes with antenatal chlamydial treatment (total studies *N* = 8).

**Study**	**Support improved adverse outcomes w/CT Tx**	**Sample size**	**Type of study**	**Intervention**	**Time of screening/Time of Tx intervention**	**CT status/Testing method**	**Outcome**	**Comments**
Martin et al. (50) New York, Louisiana, Oklahoma, Texas, Washington, US 1997	Yes	13,750 with 414 randomized	Double blind, placebo controlled RCT	Erythromycin 333 mg TID × 6 wks vs. placebo	23–29 wk/tx until 35 wk	CT/cervical cx Also eval tx for Ureaplasma, GBS	Initial analysis saw limited tx effect on LBW (8 vs. 11%, *p* = 0.4) or preterm delivery (13 vs. 5%, *p* = 0.7) Sub-analysis conducted of high persistence CT among placebo tx sites. Afterwards sig reduction in LBW 8% (9/114) vs. 17% (18/105), *p* = 0.04 No sig difference in PROM, preterm delivery, stillbirth, or neonatal deaths	20% on erythromycin remained CT pos and 46% of placebo cases with high clearance of CT. Site variability of CT persistence among placebo cases (83–89% vs. 24–25%). No tx effect seen with Ureaplasma and GBS trial either
Gray et al. (28) Rakai, Uganda 2001	Yes	4,033	Cluster RCT	Azithromycin 1 g, cefixime 400 mg, metronidazole 2 g vs. no tx except MVI and syphilis referral (eval/tx)	Various	CT/urine LCR Random sampling only Also Trich, BV, NG, HIV testing	Reduction in neonatal death (RR, 0.83; 95% CI, 0.71–0.97), LBW (RR, 0.68; 95% CI, 0.53–0.86), and preterm delivery (RR, 0.77; 95% CI, 0.56–1.05) with STI tx Reduction in general infant ophthalmia (RR, 0.37; 95% CI, 0.20–0.70); CT ophthalmia (RR 0.44, 95% CI 0.18–1.1) with STI tx	Presumptive STI tx for CT, NG, chancroid, trich, BV, and syphilis. If syphilis+, also tx with benzathine PCN G No effect on perinatal HIV transmission
Cohen et al. (51) Ohio, US 1990	Yes	338	Obs/Retro	Erythromycin 2 g × 7 days	Both 1st prenatal visit	Only CT/cervical Cx	Persistent infection vs. successful tx demonstrated improvement in LBW (3,002 vs. 3,202 g, *p* = 0.004); SGA (25.3 vs. 13.1%, *p* = 0.001), OR 0.45 (95% CI 0.23–0.88); premature contractions (24.1 vs. 4.1%, *p* = 0.00001), OR 0.13 (95% CI 0.06–0.33); PROM (20.3 vs. 7.4%, *p* = 0.0019), OR 0.31 (95% CI, 0.14–0.69); and preterm delivery (13.9 vs. 2.9%, *p* = 0.00002), OR 0.16 (95% CI, 0.06–0.47)	Group 1: CT pos successful tx Group 2: CT pos persistent/recurrent infection Group 3: matched control CT neg
Ryan et al. (52) Tennessee, US 1990	Yes	11,544 with 2,433 CT pos	Obs/Retro	Erythromycin 500 mg QID × 7 days Alternative if SE or allergy used sulfisoxazole	1st prenatal visit/tx 2nd visit	Only CT/cervical cx	CT untx vs. CT tx demonstrated improvement in PROM (5.2 vs. 2.9%, *p* < 0.001) with OR 1.79, *p* < 0.01; LBW (19.6 vs. 11%, *p* < 0.0001); infant mortality (2.4 vs. 0.6%, *p* < 0.001), OR 2.21 (0.89–5.5) borderline sig	Group 1: CT pos untx Group 2: CT pos tx Group 3: CT neg
Rastogi et al. (53) New Delhi, India 2003	Yes	350	Obs/Prosp	Erythromycin 500 mg QID × 7 days	Both 1st−3rd trimester (most tx 2nd trimester)	CT/cervical DFA, PCR Also NG, candida, BV, trich, syphilis testing	Tx group vs. untx group: higher mean gestational age (35.5 vs. 33.1 weeks, *p* < 0.05) and higher BW (2,200 vs. 2113.3 g, although not significant). Higher stillbirths untx vs. CT neg vs. CT tx groups (11.5 vs. 4.7 vs. 0%)	Group 1: pos tx Group 2: pos untx (i.e., those lost to follow-up) Group 3: CT neg
McGregor et al. (54) Colorado, US 1990	Yes	229	Double blind placebo controlled RCT	Erythromycin 333 mg TID × 7 days vs. placebo	Both 26–30 wks	CT/cervical Cx Also genital microflora testing: BV, trich, NG, *M. hominis, U. urealyticum, G. vaginalis*, Staph aureus, *Strep* sp.	PROM dec in tx group vs. placebo (6 vs. 16%, *p* < 0.01); (RR 0.4, 95% CI 0.2–0.8); PPROM dec (2 vs. 5%, *p* = 0.3) but NS In multivariate analysis, tx assoc with reduced LBW (OR 0.2, *p* = 0.02), PROM (OR 0.1, *p* = 0.01), and possibly preterm birth (*OR* = 0.1, *p*-0.06) CT specific analysis for tx vs. placebo groups: 0% (0/13) PROM vs. 50% (6/12) (RR 0.4, 0.2–0.8, *p* = 0.03) No diff in PTL, preterm birth, LBW by tx groups by univariate analysis; also no diff in groups for neonatal outcomes (not defined)	High % of placebo and tx group received non-protocol abx for vaginitis or UTI Tx compliance issues Ureaplasma pos women receiving erythromycin also with dec PROM Only CT compared to other genital microflora highly assoc w/preterm or PROM (OR 9, *p* = 0.05)
Andrews et al. (55) Multiple-sites NICHD Maternal Fetal Units, US 2006	No	2,470	Secondary analysis of 2 RCTs (metronidazole vs. placebo) (56, 57)	Non-protocol, non-standardized CT effective antibiotic (Erythromycin or Azithromycin); Potentially effective abx Primary: metronidazole vs. placebo for trich or BV	16 to <24 wks/various times ave 31.7 wks	CT with either BV or trichomonas/CT urine LCR	NS difference in spontaneous preterm birth for those without tx compared to effective or potentially effective CT tx (*p* = 0.1, *p* = 0.5) for CT pos group	
Folger et al. (5) Ohio, US 2014	Yes	3,354	Obs/Retro	Early detection and eradication (tx not specified)	Both <20 wks	CT only/not specified Also tested for NG	Dec mod-late (M/L) preterm birth with intervent vs. ref group: 12.2 vs. 14.4%, *p* = 0.05; also for spontaneous M/L preterm birth 8.2 vs. 10.8%, *p* = 0.01, RR = 0.54 (0.37–0.80) for young women ( ≤ 19 years) NS diff for preterm birth overall between groups (15.5 vs. 16.6%, *p* = 0.42); NS for very preterm birth (*p* = 0.74), or LBW (*p* = 0.52) Mean gestational age for intervent vs. ref group (37.9 vs. 38.1, *p* = 0.048) Paradoxical increase in infant death in intervent vs. ref group (2.2 vs. 0.9%, *p* = 0.003)	Intervention (intervent) group: early detection, eradication Reference (ref) group: persistent or recurrent infection

*Please note that sample size based on number of women initially enrolled into respective studies but not necessarily reflective of number of pregnancy outcomes evaluated, so these numbers should be used with caution in any direct comparisons between studies*.

*Given limited Table space the following abbreviations were used*.

*RCT, randomized controlled trial; Obs, observational; Prosp, prospective; Retro, Retrospective; wk(s), week(s); sig, significant*.

*CT, Chlamydia trachomatis; NG, Neisseria gonorrhoeae; Trich, trichomonas, HSV, herpes simplex virus; BV, bacterial vaginosis; M. hominis, Mycoplasma hominis; U. urealyticum, Ureaplasma urealyticum; G. vaginalis, Gardnerella vaginalis; Cx, culture; LCR, ligase chain reaction; tx, treated; untx, untreated; SE, side-effects; pos, positive; neg, negative; eval, evaluation; URI, upper respiratory infection; MVI, multi-vitamin, PCN, penicillin; LBW, low birth weight; BW, birth weight; PROM, premature rupture of membranes; SGA, small for gestational age; M/L, moderate-late (32–36 wks gestation); PTB, preterm birth; intervent, intervention; ref, reference*.

#### Study Objectives and Interventions

Seven studies described how maternal chlamydial infection was diagnosed. Five used cervical specimens, with four using only cultures (50–52, 54) and one using both direct fluorescent antibody (DFA) and polymerase chain reaction (PCR) (53). The other remaining two used maternal urine samples sent for nucleic acid detection with ligase chain reaction (28, 55). For the seven studies (28, 50–55) reporting maternal chlamydial prevalence rates, the mean was 11.1% with a range of 1.1–2.7% to 21.1% (28, 52). Enrollment and chlamydial screening occurred at various times depending on the study with two at the 1st prenatal visit (51, 52), one at 16 to <24 weeks (55), one at <20 weeks (5), one at 23–29 weeks (50), one at 26–30 weeks (54), and two with non-specific enrollment between first to third trimesters [(28, 53); Table 1].

All studies provided information regarding the effect of chlamydial treatment on adverse pregnancy outcomes as one of the major outcome measures. Five of the studies (5, 50–53) focused primarily on the effects of *Chlamydia trachomatis* and pregnancy outcomes, although some included an evaluation for additional organisms: *Ureaplasma urealyticum* and *Group B Streptococcus* (GBS) in one (50) and candida, syphilis, bacterial vaginosis, trichomonas, and gonorrhea (5) in others (53). The other studies evaluated *C. trachomatis* as one of several genital infections of equal importance in various combinations including bacterial vaginosis, *Trichomonas*, and *N. gonorrhoeae* among others (28, 54, 55). Only one study also evaluated treatment of sexually transmitted infections with respect to HIV perinatal transmission [(28); Table 1].

Five studies used erythromycin as the primary treatment intervention (50–54). Another, which was an STI treatment study, used empiric combination treatment with cefixime, metronidazole, and azithromycin in place of erythromycin (28). One study evaluated the effects of antibiotics with anti-chlamydial activity such as erythromycin and azithromycin as well as those with potential activity such as penicillin, amoxicillin, and ampicillin (55). In contrast, the antibiotics used in one of the studies was not stated but eradication was implied (5). Treatment protocols using erythromycin varied per study with dosing ranging from 333 mg orally three times a day for 1 week (54) to 6 weeks (50). The other three studies used higher dosing of 500 mg orally four times a day for 7 days [(51–53); Table 1].

Timing of treatment in pregnancy differed in these studies with two occurring after the first antenatal visit with chlamydial infection diagnosis (51, 52), three at various times during pregnancy (28, 53, 55), one later during pregnancy sometime between 26 and 30th weeks (54), another for a prolonged period of at least 6 weeks between 23–29th weeks until the 35th week (50), and the last unspecified (5). Only three studies recommended partner treatment (28, 50, 53), and only two of these treated partners directly [(28, 53); Table 1].

Three studies (28, 50, 54) were designed as randomized controlled trials, two (50, 54) of which were double-blinded evaluations of erythromycin vs. placebo. The other (28) was a cluster randomization study designed to evaluate interventions of empiric STI combination treatment vs. prenatal vitamins, which also included syphilis evaluation and treatment, on HIV transmission and pregnancy outcome. Of note, both of the other randomized controlled trials evaluated the effect of the erythromycin treatment intervention with respect to chlamydial infections and other genital flora/infections and pregnancy outcomes (50, 54). The remaining five studies were observational studies (5, 51–53, 55).

These studies varied in design and objective. One study, which used case-matched controls for sociodemographic factors, included three groups that compared those with chlamydial infection responsive to treatment with erythromycin, those unresponsive to treatment with persistent infection, and those that were uninfected (51). Another compared pregnant women initially screened for chlamydia and untreated (even if positive) with those that were later screened and treated with erythromycin if they had tested positive (52). Another was a prospective study evaluating the effects of erythromycin on chlamydial infection in pregnant women, and the group that was infected and untreated was composed of women initially lost to follow-up (53).

In contrast, one of the studies was a secondary analysis of several parent studies (56, 57) that were randomized controlled trials evaluating the effects of metronidazole for bacterial vaginosis and trichomonas on adverse birth outcomes; the study analyzed the impact of antibiotics on chlamydial infection and infant outcomes as a secondary aim (55). The last one was a retrospective cohort using linked public health databases to evaluate birth outcomes for pregnant women with early chlamydial infection and unspecified treatment eradication vs. those with recurrent and persistent infection [(5); Table 1].

#### Adverse Pregnancy Outcomes

Seven of eight studies provided some support regarding the potential benefits of chlamydial screening and treatment during pregnancy to prevent adverse pregnancy outcomes such as preterm delivery, premature rupture of membranes, and low birth weight (5, 28, 50–54). Five of these studies provided direct support of the benefit of treatment with erythromycin in reduction of adverse outcomes [(50–54); Tables 1, 2]. In contrast, the study by Andrews et al., found that chlamydia-infected pregnant women treated with effective or potentially effective antibiotics against chlamydia did not show differences in rates of preterm delivery from those of untreated women (*p* = 0.11 and *p* = 0.5); no other adverse pregnancy outcomes were evaluated (55).

**Table 2:** Studies of prevention of adverse pregnancy outcomes with antenatal chlamydial treatment (*N* = 8).

**Study**	**Support improved adverse outcomes w/CT Tx**	**Type of study**	**Direct or indirect evidence**	**Significant adverse pregnancy outcomes findings**						**Other**
				**Preterm**	**PROM**	**LBW**	**SGA**	**Neonatal death**	**Still birth**	
Martin et al. (50)	Yes	RCT	Direct	NS^**^	NS	17 to 8%, *p* = 0.04 (53% reduction for tx group) special analysis	–	NS	NS	Initial analysis no LBW effect until restricted to study sites w/high persistence CT among placebo women^**^
Gray et al. (28)	Yes	RCT mass tx	Indirect mass tx (not CT specific reductions)	RR 0.77; 95% CI, 0.56–1.05; borderline sig Note: used preterm ≤ 36 wks (more restrictive definition)	NS	RR 0.68 (0.53–0.86)	–	RR, 0.83 (0.71–0.97)	NS	Dec ophthal: RR 0.37 (0.2–0.7); dec specific to CT ophthal: 1.1–0.6% (RR 0.44, 0.18–1.1); no effect on HIV MTCT
Cohen et al. (51)	Yes	Retro/Obs with case-match controls 3 groups: CT tx; CT persistent; CT neg	Direct (diff between CT tx and persistent infection)	Persistent CT vs. successful CT tx groups						
				13.9 vs. 2.9%, *p* = 0.00002; OR 0.16 (0.06–0.47)	20.3 vs. 7.4%, *p* = 0.0019), OR 0.31 (95% CI, 0.14–0.69) Also: premature contractions 24.1 vs. 4.1%, *p* = 0.00001; OR 0.13 (0.06–0.33)	3,002 vs. 3,202 g, *p* = 0.004; OR 0.31 (0.14–0.69)	25.3 vs. 13.1%, *p* = 0.001, OR 0.45 (0.23–0.88)	–	NS	NS diff for antepartum hemorrhage and for PPE
Ryan et al. (52)	Yes	Retro/obs 3 groups: CT tx; CT untx; CT neg	Direct (diff between CT untx vs. CT tx)	Untx CT vs. CT tx groups						
				Not reported for prematurity or preterm labor	5.2 vs. 2.9%, *p* < 0.001; OR 1.79, *p* < 0.01	19.6 vs. 11%, *p* < 0.0001	–	Survival 97.6 vs. 99.4%, *p* < 0.001, OR 2.21 (0.89–5.49) *p* < 0.08 (borderline sig)	–	Note that non-survivors included stillbirth cases
Rastogi et al. (53)	Yes	Obs 3 groups: CT tx; CT untx; CT neg	Direct (diff between CT tx vs. untx)	Untx CT vs. tx groups						–
				33.1 vs. 35.5 wks gestational age, *p* < 0.05	–	BW 2113.3 g vs. 2,200 but NS	–	–	11.5 vs. 0% (no *p* = value)	
McGregor et al. (54)	Yes	RCT	Direct and Indirect (Tx not just CT directed but genital microflora)	Borderline dec in MVA only with tx vs. placebo OR = 0.1, *p* = 0.06	PROM dec in tx vs. placebo 6 vs. 16%, *p* < 0.01; RR 0.4 (0.2–0.8); (OR 0.1, *p* = 0.01) in MVA PPROM dec 2 vs. 5%, *p* = 0.3, but NS Tx with dec PROM CT specific tx vs. placebo: PROM 0% (0/13) vs. 50% (6/12), RR 0.4 (0.2–0.8), *p* = 0.03	Dec LBW (OR 0.2, *p* = 0.02) with tx vs. placebo in multivariate analysis	–	–	–	NS diff groups for chorio, PPE, or neonatal outcomes (not defined) NS diff for LBW, PTL or preterm birth by UVA Only CT highly assoc w/preterm or PROM (OR 9, *p* = 0.05)
Andrews et al. (55)	No	Secondary analysis	None and also indirect (since secondary analysis for CT, BV and trich)	NS (No diff for tx with CT effective, potentially effective, or no effective abx, *p* = 0.1, *p* = 0.51)	–	–	–	–	–	–
Folger et al. (5)	Yes	Obs	Indirect (but tx not specified)	NS diff overall PTB or very PTB with intervent, *p* = 0.42, *p* = 0.74 Dec M/L PTB intervent vs. ref: 12.2 vs. 14.4%, *p* = 0.05; spont M/L PTB, 8.2 vs. 10.8%, *p* = 0.01; RR 0.54 (0.37–0.80) for young women	–	NS diff overall LBW, *p* = 0.52	–	2.2 vs. 0.9%, *p* = 0.003 (higher in intervent vs. ref group) paradoxical effect	–	–

–*, not evaluated; NS, findings not significant; CT, Chlamydia trachomatis; Tx, treated; Untx, untreated; pos, positive; neg, negative; PTB, preterm birth; M/L, moderate to late preterm birth (32–36 wks gestation); LBW, low birth weight; PROM, premature rupture of membranes; SGA, small for gestational age; RR, rate ratio; RCT, randomized controlled trial; PPE, post-partum endometritis; ophthal, ophthalmia; MVA, multivariate analysis; UVA, univariate analysis; intervent, intervention group; ref, reference group*.

** *refers to comment in the “other” column*.

#### Premature Rupture of Membranes, Preterm Delivery, Low Birth Weight, Small for Gestational Age, and Neonatal Death

Three of five studies (28, 50–52, 54), evaluating premature rupture of membranes after a treatment intervention, reported reduction in this specific outcome. A reduction in premature rupture of membranes was seen in some of the studies when comparing untreated or persistently infected vs. treated women: 5.2 to 2.9% (OR 0.56, 95% CI 0.37–0.85) (52), 20.3 to 7.4% (OR 0.31, 95% CI 0.14–0.69) (51), and 50 to 0% (RR 0.4, 95% CI 0.2–0.8) [(54); Table 2].

Seven (5, 28, 50, 51, 53–55) of eight studies, evaluated the effect of maternal treatment on preterm delivery, and all but two studies (50, 55) suggested a possible benefit in preventing this outcome. The strongest evidence reported a significant reduction from 13.9 to 2.9% (*p* = 0.00002) in preterm births for 244 chlamydia-infected women receiving treatment compared to 79 of those persistently infected, including a significantly decreased odds of delivering a preterm infant if treated (OR 0.16, 95% CI, 0.06–0.47) (51). Another study suggested higher mean gestational ages by more than 2 weeks (35.5 vs. 33.1 weeks, *p* < 0.05) for women treated for chlamydia compared to untreated women (53). The remaining studies provided either more indirect evidence of the benefits of treatment for preterm delivery (54), mixed results depending on the specific analysis (5), or evidence of borderline significance [(28); Table 2].

Seven studies evaluated the impact of chlamydial treatment in pregnancy on infant birth weights (5, 28, 50–54). Three studies provided more direct support for improvement in birth weights or reduction in low birth weight infants with chlamydial treatment in pregnancy (50–52). One of these studies reported a significant increase in mean birth weight by 200 g [*p* = 0.0041; (51)]. Two other studies found significant reductions in low birth weight infants with maternal treatment: 17 to 8% [*p* = 0.04; (50)] and 19.6 to 11% [*p* < 0.0001; (52)]. Other studies found indirect support of the benefit of these interventions (28, 54), whereas two studies did not find significant differences in the number of low birth weight infants (5, 53). Of note, one of the studies that found reductions in preterm delivery and low birth weight infants also noted that treated women were also less likely to deliver infants who were small for gestational age (OR 0.45, 95% CI 0.23–0·88) [(51); Table 2].

Only four studies evaluated neonatal survival following treatment during pregnancy (5, 28, 50, 52). Findings were mixed with one study (5), which showed a paradoxical increase in neonatal deaths from 0.9 to 2.2% (*p* = 0.003) following a treatment intervention, while other studies suggested a possible decline or no significant difference in neonatal mortality (50). In contrast, four studies (28, 50, 51, 53) evaluated differences in stillbirth rates, but only one found a decrease in this outcome with maternal chlamydial treatment [(53); Table 2].

#### Study Quality

These studies analyzed varied widely with respect to study design, specimens collected, method of testing, timing of testing, evaluation for other STIs, and type of antibiotic and regimen used for treatment. As a result, each had strengths and limitations regarding study quality. Since only 3 studies (28, 50, 54) were randomized, selection bias may have been a factor that impacted the other study results (5, 51–53, 55), particularly since many of the adverse pregnancy outcomes in question may be influenced by multiple factors beyond infectious etiologies such as *C. trachomatis*.

Other issues included heterogeneity of methods used to test for *C. trachomatis*, a limited number of studies which also involved treatment of partners (28, 53), and only some employed repeat testing or test of cure after treatment to evaluate for treatment failure (50–54). Consistent with the practices of the time these studies were conducted, the majority used culture to diagnose chlamydial infection, which are less sensitive that nucleic acid amplification testing (NAAT) methods currently employed. Although one study employed directly observed antibiotic therapy (28), the impact of screening and treatment in other studies may have also been influenced by patient follow-up and treatment non-compliance, which were high in certain studies (50, 53, 54). Other issues included persistence of chlamydial infection in spite of treatment and spontaneous clearance of infection in placebo cases in one study (50). Few studies (28, 55) also commented on whether other antibiotics were taken by women for other reasons during pregnancy (50, 54), which may also have had an impact on *C. trachomatis* clearance and pregnancy outcomes.

### Studies Preventing Neonatal Chlamydial Infection

#### Study Characteristics

Only seven studies (39, 49, 58–62) provided data regarding the effect of screening and treatment in preventing neonatal chlamydial infection. All seven studies were written in English, and all studies occurred in the U.S., with the exception of one from Quebec, Canada (49). Two studies reported that their cohorts consisted primarily of young black women (59, 61), and two others were mainly composed of young, Hispanic and black women (58, 60). None of the studies were published in the last 15 years, ranging in publication date from 1982 to 1994. Cohort sizes varied based on study objectives, ranging from 21 to 1,082 women [(39, 62); Table 3].

**Table 3:** Prevention of adverse neonatal outcomes with antenatal chlamydial treatment (total studies *N* = 7).

**Study**	**Support improved adverse outcomes w/CT Tx**	**Sample size**	**Type of study**	**Intervention**	**Time of screening/** **Timing of intervention**	**CT status/** **Testing method**	**Infant outcomes**	**Comments**
Schachter et al. (58) California, US 1986	Yes	184	Prosp/Obs	Erythromycin 400 mg QID × 7 days	1st prenatal visit/36 wks	CT only/cervical Cx	For CT tx vs. control (untx) group, CT infant infection dec 50% (12/24) to 7% (4/59), *p* < 0.001. Infant control group: 12 seropositive infants (1 conjunctivitis, 4 pneumonia, 7 pos cx); infant tx group: 4 seropositive (2 pneumonia, 1 conjunctivitis, 3 with pos CT cx)	High loss of mothers and infants in follow-up Controls = women CT pos but refused tx
Alary et al. (49) Quebec, Canada 1994	Yes, somewhat (indirect)	210	Double blind RCT	Amoxicillin 500 mg PO TID vs. Erythromycin 500 mg QID × 7 days	1st prenatal visit/ <38 wks	CT only/cervical and urethral Cx	0% (0/152) infants with CT pos cx in tx groups	No vertical transmission in tx groups No untx group
FitzSimmons et al. (59) Pennsylvania, US 1986	Yes, somewhat (mostly indirect)	221	Prosp/Obs	Erythromycin 500 mg PO QID × 10 days	<21 wks/36 wks	CT only/cervical Cx	0% (0/16) infants with CT of tx group; 66.7% (2/3) infants with CT untx group	High loss to follow-up Infants of CT pos tx; CT neg (controls); small group CT pos untx (3)
Crombleholme et al. (60) California, US 1990	Yes, somewhat (indirect)	193	Open CT (no randomization)	Amoxicillin 500 mg TID vs. Erythromycin 500 mg QID × 7 days	1st prenatal visit/36 wks	CT only/cervical Cx	5.1% (2/39) amoxicillin vs. 11.1% (4/36) erythromycin infants with CT, but NS diff Low rates vertical transmission in tx groups	High loss to infant follow-up No untx group
Black-Payne et al. (61) Louisiana, US 1990	Yes, somewhat (indirect)^**^	199	Prosp/Obs	Evaluate Chlamydiazyme; Erythromycin 500 mg QID × 7 days offered if CT pos	Both 28–32 wks	CT/Chlamydia-zyme rapid EIA-also NG screen	NS diff between infants of women CT neg (48) and CT pos (50) tx for conjunctivitis or respiratory tract illness	Also some info on pregnancy outcome-no diff in ROM, preterm birth CT pos group presumed tx ^**^Expect no diff (CT pos tx vs. CT neg) for infants if tx was effective
McMillan et al. (62) New York, US 1985	Yes	1,082	Prosp/Obs	Erythromycin tx not standardized	32–36 wks/ not standardized	CT/cervical cx-also info on NG, syphilis, condylomata acuminata, HSV testing/eval	0% (0/16) infants with CT in tx group; 23.8% (5/21) with CT in untx group, *p* < 0.04 Some infant disease (4 conjunctivitis, 1 pneumonia requiring hospitalization) in CT pos infants from untx group No diff in otitis media, URIs in first 6 months	Compliance unknown Limited infant follow-up
Bell et al. (39) Washington, US 1982	No	21	Double blind placebo controlled RCT	Amoxicillin 500 mg TID × 10 days vs. placebo	Both 24 wks	CT only/cervical cx	No sig diffs in infant outcomes 37.5% (3/8) infants CT pos for tx group vs. 33.3% (1/3) for placebo group (no *p-*values listed)	Infants all received silver nitrate eye ppx at birth; all with conjunctivitis received PO erythromycin Limited numbers; 6/21 lost to follow-up or later excluded 3/9 tx women later with pos post-partum cx; 4/6 placebo tx with pos post-partum cx Infant outcomes only reported for vaginal deliveries

*Sample size based on number of women initially enrolled into respective studies but not necessarily reflective of number of infant outcomes evaluated or the number that were found to have CT infections*.

*Please also note that untreated and treated in outcomes refers to women with CT diagnosed in pregnancy*.

*CT, Chlamydia trachomatis; NG, Neisseria gonorrhoeae; HSV, herpes simplex virus; Cx, culture; CT only, refers to CT as only infection tested for during study; Tx, treated; Untx, untreated; pos, positive; neg, negative; URI, upper respiratory infection; diff, difference; NS, no(t) significant; ppx, prophylaxis; sx, symptomatic or symptoms; eval, evaluation*.

** *refers to comments in the comment section for this row*.

#### Study Objectives and Interventions

The primary focus of all of these studies was *Chlamydia trachomatis* as opposed to other sexually transmitted infections or genital infections. Only two studies collected some information on other STIs apart from chlamydia (61, 62). All seven studies used cervical specimens to screen for *Chlamydia trachomatis* infection in their cohorts of pregnant women. Six (39, 49, 58–60, 62) of seven studies used cervical cultures, of which one also used urethral swabs for culture (49). One of the studies used a chlamydia rapid enzyme immunoassay antigen detection assay (Chlamydiazyme) to assess for chlamydial infection (61). For the four studies reporting chlamydial prevalence rates, findings ranged broadly from 1.7 to 26% (49, 59, 61, 62). Three studies (49, 58, 60) conducted chlamydial testing of pregnant women at their first prenatal visit, while others provided screening later [<21 weeks gestation (59), 24 weeks (39), 28–32 weeks (61), and 32–36 weeks (62); Table 3].

Erythromycin was used as either the main therapeutic intervention or one of the interventions in six of seven studies (49, 58–62). In contrast, one study used only amoxicillin as treatment (39). The dosage of erythromycin varied from 400 mg given orally four times a day for 7 days (58) to 500 mg orally four times a day for seven (49, 60, 61) to 10 days (59). Information regarding the dosage of erythromycin was not discussed in one study due to lack of standardization, which was based upon the discretion of individual practitioners (62).

Four studies were prospective with erythromycin used as the therapeutic intervention (58, 59, 61, 62). Two studies were treatment trials comparing amoxicillin to erythromycin, which was the standard of care at the time (49, 60). One of these studies was a double blind, randomized trial of amoxicillin vs. erythromycin (49). The other was an open trial of amoxicillin and erythromycin, where all participants were offered amoxicillin as an alternate treatment option to erythromycin (60). While the main objective of these two studies was to provide a comparison of side-effects between treatment groups, perinatal chlamydial infection was one of the outcome measures evaluated (49, 60). The focus of the last study differed from the others; it was a double blind, randomized study of amoxicillin vs. placebo (39). In each of the studies, treatment occurred at different time points during pregnancy. Treatment in three studies occurred at 36 weeks (58–60), but occurred at various times in the other studies: 24 weeks (39), 28–32 weeks (61), <38 weeks (49), or not specified [(62); Table 3].

#### Infant Outcomes

Only three of the studies primarily focused on determining the potential of chlamydial treatment to prevent perinatal chlamydial infection by comparing infants of women with chlamydial infections, who were either treated or untreated (39, 58, 62). As a result, these studies were able to provide more direct information about the potential to reduce perinatal chlamydial infections with maternal treatment during pregnancy. Of note, another study also provided information on perinatal chlamydial infection in untreated infants, but the number was too small (only three infants) to draw any definitive conclusions (59). In one of the studies, 32 women refused treatment after all women with chlamydial infection were offered erythromycin; 24 infants of untreated women served as controls for the 59 infants of treated women (58). In contrast, the decision to treat or not treat in another study was based on provider discretion; they followed 21 infant outcomes from a group of 47 chlamydia-infected, untreated women and outcomes of 16 infants from 38 women treated with erythromycin (62). The last study, which sought an alternative to erythromycin for treatment of chlamydia, randomized 21 women to treatment with either amoxicillin or placebo and followed infant outcomes [(39); Table 3].

Infant chlamydia infection was detected by different methods in the studies. Two studies were the most comprehensive using a combination of methods including persistence of chlamydia IgG antibody in first year of life, chlamydia mucosal cultures (nasopharynx, conjunctiva, rectum), and evaluation for symptomatic infection such as pneumonia and conjunctivitis (58, 60). A similar strategy was also used in another study that tested infant tears and blood for chlamydia antibody and obtained chlamydia cultures (nasopharynx, oropharynx, conjunctiva, rectum, genitalia) (39). Two other studies used a combination of infant mucosal cultures (nasopharynx and conjunctiva) taken from 2 to 11 or 12 weeks along with evaluation for symptomatic disease such as conjunctivitis and pneumonia among others (59, 62). For the remaining studies, one used only surface cultures collected 1 week after birth (49), and another evaluated exclusively for symptomatic disease in the first 6–8 weeks of life [(61); Table 3].

Two studies found significantly decreased rates of infant chlamydial infection among those born to women receiving treatment with erythromycin as opposed to no treatment (58, 62). A decrease in infant chlamydial infection from 50% (12/24) to 7% (4/59) (*p* < 0.001) and 23.8% (5/21) to 0% (0/16) (*p* < 0.04) were noted with treatment (58, 62). Symptomatic chlamydia infection with conjunctivitis and pneumonia were more frequently seen among infants of untreated women (58, 62). Two other studies reported observations on small numbers of infants, who were born to untreated women: 2 of 3 infants had positive cultures (59), and 1 of 2 infants had conjunctivitis among women who refused completion of erythromycin [(60); Table 3].

Other studies provided indirect evidence about the potential to prevent adverse neonatal outcomes through maternal treatment, as low rates of infant chlamydial infection and symptomatic infant disease were observed among treated women. These studies primarily evaluated infant outcomes for treated women (49, 59, 60), but one study compared differences in infant outcomes for treated, chlamydia-infected women vs. chlamydia-uninfected women (61).

In infants of treated women, they found low rates of infant chlamydial infection based on either chlamydial mucosal cultures or persistent antibody levels ranging from 0% (0/152, 0/16) to 11% (4/36) (49, 59, 60); another study found no significant differences in symptomatic disease among infants born to women without chlamydial infection vs. those with treated chlamydial infection, which would be the expected outcome with effective chlamydial treatment [(61); Table 3]. In contrast to the other six studies, one small study did not find significant differences in the number of neonatal chlamydial infections among women treated with amoxicillin vs. placebo [3/8 (37.5%) vs. 1/3 (33.3%)] (39).

#### Study Quality

Some factors that could have impacted the quality of results included test of cure to ensure the therapeutic intervention had eradicated chlamydial infection, maternal compliance with the treatment regimen, loss of follow-up, and cohort sample size. In five of seven studies, information regarding effectiveness of treatment was documented by test of cure (i.e., repeat cultures after treatment), which showed no evidence of continued infection in most treated women (92–99.5%) (49, 58, 60, 61). The exception was one study that used amoxicillin, which had much higher failure rates of 33.3% (3/9) (39).

Information regarding non-compliance with the study such as maternal follow-up and treatment recommendations also varied considerably from 5.2% to as high as 41.8% (39, 49, 58–61). These findings were particularly striking for the limited degree of infant follow-up achieved by these studies, which ranged from 38.9 to 72.4% (39, 49, 58–61). Other possible confounders that may have impacted infant outcomes included limited information on testing of sexual partners, which was only done in one study (49), and treatment of sexual partners, which was offered in five of the studies, but no information was available in any of the studies regarding the percentage of partners that actually received treatment (39, 49, 58, 60, 61). While delivery method (caesarian section vs. vaginal delivery) could also impact the likelihood of chlamydia vertical transmission, few of the studies excluded infants that were born via caesarian section without prior rupture of membranes (39, 60). Other factors that could have impacted results included the reliance on infant chlamydia serology along with cultures to diagnose chlamydial infection (39, 58, 60). which were the main methods used at the time these studies were conducted but lack the sensitivity and specificity of molecular testing methods (NAATs) currently used. In addition, one of the studies (53) also used Chlamydiazyme, an enzyme immunoassay, to identify maternal chlamydial infections. This method of testing has been reported to have decreased sensitivity in the detection of chlamydial infections compared to culture (63). There was also a lack of information regarding how symptomatic infections such as pneumonia and conjunctivitis were determined to be due to chlamydia as opposed to other etiologies (58).

## Discussion

When viewed comprehensively, this review provides fair to moderate support from thirteen of fifteen studies that chlamydial screening and treatment in pregnancy may lead to improved pregnancy and infant outcomes. However, the heterogeneity of those studies precluded any combined quantitative assessments such as meta-analyses of the effects of treatment on those outcomes. Overall, the strength of the evidence was limited by only a handful of studies directly comparing pregnancy or neonatal outcomes between treated and untreated chlamydia-infected mothers. As with any focused review, there was likely some degree of publication bias given the inherent difficulty in locating studies with negative findings (Tables 1–3).

Three studies, which included two retrospective/observational studies and one double-blind randomized placebo controlled trial, provided the strongest evidence within the group suggesting that chlamydial treatment with erythromycin may lead to improved pregnancy outcomes such as reduction in preterm birth, premature rupture of membranes, and/or low birth weight infants (50–52). These studies also had important limitations. This included: (1) The regimen of erythromycin used in one (50); (2) Significant findings only after adjustment of one of the study's sample size because of spontaneous clearance of infection in placebo cases (50); (3) Evaluation of the effect of treatment on other infections such as *Ureaplasma* and *Group B Streptococcus* apart from just *C. trachomatis* (50); (4) Use of persistent or recurrent infection as opposed to untreated patients in one study (51); (5) Lack of information regarding use of other antibiotics during pregnancy with possible effects on chlamydial infection (50–52).

In contrast, the strongest evidence that antenatal chlamydial treatment with erythromycin may decrease neonatal infection came from two observational studies in the mid-1980s (58, 62). Both studies, however, had several limitations including significant losses to follow-up and use of a non-standardized treatment regimen in one study [(58, 62); Tables 1–3].

Viewed collectively, our review of these studies also highlights serious gaps in existing knowledge. While the study by Martin et al. came the closest, none of the randomized controlled trials focused exclusively on the effect of early screening and treatment of chlamydial infections by comparing treated and untreated groups with respect to adverse pregnancy and neonatal outcomes (50). Furthermore, only a few studies used sensitive molecular methods such as PCR to detect *C. trachomatis* infection, and none of the studies focused exclusively on evaluating more easily administered antimicrobials such as single dose azithromycin as the only intervention. Additional gaps include the lack of interventional research in regions of the world outside of the US, where STIs like *C. trachomatis* are most prevalent and the burden of problems such as preterm birth and adverse neonatal and pregnancy outcomes are the highest (3, 4, 29). In fact, only two of the studies meeting selection criteria for review were in such countries [(28, 53); Tables 1–3].

Although countries such as the U.S. have implemented chlamydial screening for pregnant women, particularly for those at risk for the past few decades, many countries around the world, particularly resource-limited countries have continued to rely on the WHO-endorsed “syndromic approach” to symptomatic STIs in pregnancy (1, 3, 29, 64–66). Few studies have evaluated the prevalence of *Chlamydia trachomatis* in pregnancy in high-risk regions of the world, and the extent of the morbidity associated, particularly infant morbidity, is largely unknown (3, 4). Given the greater recognition in recent years of the association of preterm birth with worldwide morbidity and mortality, the possible role that chlamydial infections in pregnancy may play and the potential to treat these infections assumes even greater importance (3, 67).

## Conclusion

This focused review has found fair to moderate evidence with a consistent trend supporting a potential beneficial role for screening and treating *Chlamydia trachomatis* in pregnancy in order to reduce adverse pregnancy outcomes such as premature rupture of membranes, premature, and low birth weight infants and neonatal chlamydial infection. However, further research, is needed to optimize understanding and benefits of such interventions, particularly with regards to adverse pregnancy outcomes in regions of the world most at risk.

## Author Contributions

KA and JK have collaborated to perform the literature search, article selection, and the writing and development of this manuscript. KN-S has assisted with the analysis, writing, development, and editing of this manuscript. All authors contributed to the article and approved the submitted version.

## Conflict of Interest

The authors declare that the research was conducted in the absence of any commercial or financial relationships that could be construed as a potential conflict of interest.

## Footnotes

^1^Please note that Gray et al. (28), study also discusses neonatal ophthalmia but has been included in adverse pregnancy outcomes section because it primarily deals with adverse pregnancy outcomes as opposed to neonatal outcomes.
