# Supplementary material for: Chlamydia trachomatis Screening and Treatment in Pregnancy to Reduce Adverse Pregnancy and Neonatal Outcomes: A Review
Source: Front Public Health. 2021 Jun 10;9:531073. doi: 10.3389/fpubh.2021.531073 (PMC8222807; doi:10.3389/fpubh.2021.531073)
Supplement: Supplementary file 1 [file Table_1.docx]

| **ADDITIONAL SUPPLEMENTAL TABLE 1: SOME NOTABLE EXCLUDED STUDIES OF PREVENTION OF ADVERSE PREGNANCY AND NEONATAL OUTCOMES WITH ANTENATAL CHLAMYDIAL TREATMENT (TOTAL STUDIES N= 8 )** | | | | | | | | |
| --- | --- | --- | --- | --- | --- | --- | --- | --- |
| **Study** | **Support Improved Adverse Outcomes w/ CT tx** | **Sample Size** | **Type of Study** | **Type of Intervention** | **Time of Screening/ Timing of Intervention** | **CT Status/ Testing Method** | **Outcome** | **Comments/**  **Rationale for**  **Exclusion** |
| **Cochrane Review**^42^  **2000**  **[Included studies by Adair 1998, Alary 1994, Alger 1991, Bell 1982, Bush 1994, Edwards 1996, Magat 1993, Martin 1997, Rosenn 1995, Silverman 1994, Turrentine 1995]**^39,49,50, 68-74^ | Not applicable | Variable | Systematic review of 11 RCTs  (Includes Martin, Alary, Bell studies featured in Tables 1-3) | Variable | Variable | CT only | Only 1 study (Martin) looked at tx effect on adverse pregnancy outcomes  Only 2 studies included (Alary and Bell) included neonatal CT cx (ie neonatal outcomes)  Other RCTs included did not evaluate effect of tx on adverse pregnancy or neonatal outcomes | Only 3 individual studies with adverse pregnancy or neonatal outcome data |
| **French**^45^  **Colorado, US**  **2006** | Yes, somewhat but indirect | 1105 | Secondary analysis of 4 studies^45,54,75-77^ | Non-specific tx (not clearly defined) | Various times: mean 24 wks, 26-30 wks, 16-26 wks, 1^st^ prenatal visit/ tx time not clearly defined | CT alone or in combination with BV, trichomonas/ cervical Cx or EIA  Also info on *Mycoplasma hominis,* NG | Preterm birth reduced with tx (RR 0·16, 95% CI 0·04-0·66)  Subset analysis of 1 prospective trial[^79^](#_ENREF_79): preterm birth 2.4% Tx of black women vs control 18% RR 0·13 (95% CI 0·02-1, p=0·02)  *not CT specific and that trial was looking for tx response for BV | No CT specific analysis with regards to adverse pregnancy outcomes |
| **Kovacs**^46^  **Hungary**  **1998** | Unclear | 6161 | Prosp/Obs | Roxithromycin  150mg BID x 10 days | Unclear when screening intervention occurred; 3 of 7 centers treated CT-pos patients | CT ectocervix specimens tested by Gen-Probe | No sig correlations btw LBW, PROM, dysmaturity and CT infection  In premature labor (8% vs 5%, p<0·05), combined LBW and perinatal death (17·5% vs 5·5%, p<0·05), CT significantly infection higher  17 CT pos tx with normal delivery in 14, premature delivery in 2 (1 with incomplete tx), and spontaneous abortion in 1 (that had incomplete tx) | No specific statistical analysis of treated versus untx groups; size of tx group evaluated too small to determine findings |
| **Rivlin**^47^  **Mississippi, US**  **1997** | No | 1350 | Prosp/Obs | Erythromycin | Both 1^st^ prenatal visit | CT cervical Ag | Tx (23) vs untx (58) PROM 5% vs 5%; preterm delivery 15% vs 12%; stillbirth 4% vs 0%; LBW 20% vs 14%; conjunctivitis 28% vs 6%; pneumonia 14% vs 6%  *only 1 of the tx group cases were positive for CT conjunctivitis | No direct comparisons of data to interpret significance of findings |
| **Jain**^43^  **Georgia, US**  **1999** | Unclear | 55 | Retro/Obs | Unknown | Unknown | Unknown | For mothers of CT-infected infants with conjunctivitis and/or pneumonia:  12/55 (22%) no prenatal CT testing  19/55 (35%) had CT pos in pregnancy or at delivery and not tx  16/55 (29) women neg during pregnancy  8/55 (14%) tx for CT in pregnancy but not retested | Excluded based on unclear outcomes |
| **Nadafi**^44^  **Shiraz, Iran**  **2005** | No, also unclear | 92 | RCT | Amoxicillin 500mg TID x 7 days vs Erythromycin 500mg QID x 7 days vs placebo | Unknown | CT serum IgM, IgG | No correlation seen between preterm labor and CT | Excluded based on unclear outcomes  No specific data on outcomes tx and untx groups (amoxicillin vs erythromycin vs placebo) |
| **Banniettis**  **New York, US**^30^  **2021** | Yes, indirect | 297 | Prosp/seroepi | Unknown | Unknown | CT serum IgG  (of children)  Maternal screening CT NAAT | Prescreening 1991-1995 children < 10 yrs (10/54) 18.5% vs postscreening 2012-2015 (0/55) 0% (p=0.0006) | Excluded given no specific infant outcomes only seroprevalence info. Lack specifics of maternal screening and treatment (charts reviewed regarding screening status for NAAT); per authors, maternal treatment with azithromycin (unpublished inquiry to authors)  Seroepi study comparing prospectively collected specimens of children 1-20 yrs from 2012-2015 and compared with left over specimens from 1991-1995 (before and after CT screening implemented) |
| **Kohlhoff**  **New York, US^31^**  **2020** | Yes, indirect | 880 | Retro/obs | Unknown | Unknown | CT conjunctival cx (infant)  No maternal screening | Prescreening period (1986-1993) 99/636 (15.6%) CT positive vs 4/228 (1.8%) post-screening period (p<0.001) | Excluded given lack of specifics of maternal screening and treatment  Retrospective study comparing CT conjunctival infant cultures before and after CT screening implemented |

*Please note that sample size based on number of women initially enrolled into respective studies but not necessarily reflective of number of infant outcomes evaluated or the number that were found to have CT infections*

Abbreviations used: CT= *Chlamydia trachomatis;* NG= *Neisseria gonorrhoeae*; HSV= herpes simplex virus; Cx= culture; Tx= treated; Untx= untreated; pos=positive; neg= negative; NAAT=nucleic acid amplification testing

RCT= randomized controlled trial; Obs=observational; Prosp=prospective; Retro=Retrospective; Seroepi=Seroepidemiologic; wk(s)= week(s); sig=significant

Additional References Only Included in Supplemental Table

68. Adair CD, Gunter M, Stovall TG, McElroy G, Veille JC, Ernest JM. Chlamydia in pregnancy: a randomized trial of azithromycin and erythromycin. *Obstet Gynecol.* 1998;91(2):165-168.

69. Alger LS, Lovchik JC. Comparative efficacy of clindamycin versus erythromycin in eradication of antenatal Chlamydia trachomatis. *American journal of obstetrics and gynecology.* 1991;165(2):375-381.

69. Bush MR, Rosa C. Azithromycin and erythromycin in the treatment of cervical chlamydial infection during pregnancy. *Obstet Gynecol.* 1994;84(1):61-63.

70. Edwards MS, Newman RB, Carter SG, Leboeuf FW, Menard MK, Rainwater KP. Randomized Clinical Trial of Azithromycin vs. Erythromycin for the Treatment of Chlamydia Cervicitis in Pregnancy. *Infect Dis Obstet Gynecol.* 1996;4(6):333-337.

71. Magat AH, Alger LS, Nagey DA, Hatch V, Lovchik JC. Double-blind randomized study comparing amoxicillin and erythromycin for the treatment of Chlamydia trachomatis in pregnancy. *Obstet Gynecol.* 1993;81(5 ( Pt 1)):745-749.

72. Rosenn MF, Macones GA, Silverman NS. Randomized trial of erythromycin and azithromycin for treatment of chlamydial infection in pregnancy. *Infect Dis Obstet Gynecol.* 1995;3(6):241-244.

73. Silverman NS, Sullivan M, Hochman M, Womack M, Jungkind DL. A randomized, prospective trial comparing amoxicillin and erythromycin for the treatment of Chlamydia trachomatis in pregnancy. *Am J Obstet Gynecol.* 1994;170(3):829-832.

74. Turrentine MA, Troyer L, Gonik B. Randomized prospective study comparing erythromycin, amoxicillin, and clindamycin for the treatment of chlamydia trachomatis in pregnancy. *Infect Dis Obstet Gynecol.* 1995;2(5):205-209.

75. McGregor JA, French JI, Jones W, Milligan K, McKinney PJ, Patterson E, Parker R. Bacterial vaginosis is associated with prematurity and vaginal fluid mucinase and sialidase: results of a controlled trial of topical clindamycin cream. *American journal of obstetrics and gynecology.* 1994;170(4):1048-1059; discussion 1059-1060.

76. McGregor JA, French JI, Parker R, Draper D, Patterson E, Jones W, Thorsgard K, McFee J. Prevention of premature birth by screening and treatment for common genital tract infections: results of a prospective controlled evaluation. *American journal of obstetrics and gynecology.* 1995;173(1):157-167.

77. McGregor JA, French JI, Richter R, Franco-Buff A, Johnson A, Hillier S, Judson FN, Todd JK. Antenatal microbiologic and maternal risk factors associated with prematurity. *American journal of obstetrics and gynecology.* 1990;163(5 Pt 1):1465-1473.
